# Prohaptoglobin inhibits the transforming growth factor-β-induced epithelial-to-mesenchymal transition *in vitro* by increasing Smad1/5 activation and suppressing the Smad2/3 signaling pathway in SK-Hep1 liver cancer cells

**DOI:** 10.1371/journal.pone.0266409

**Published:** 2022-05-17

**Authors:** Mi-Kyung Oh, Hansol Joo, In-Sook Kim

**Affiliations:** 1 Department of Medical Life Sciences, College of Medicine, The Catholic University of Korea, Seoul, Korea; 2 Bio-MAX institute, Seoul National University, Seoul, Korea; Midwestern University, UNITED STATES

## Abstract

Transforming growth factor-β (TGF-β) is an important inducer of the epithelial-to-mesenchymal transition (EMT) in various cancers. Our previous study demonstrated that prohaptoglobin (proHp) stimulates Smad1/5 activation *via* ALK1, a TGF-β type I receptor, in endothelial cells, suggesting that proHp plays a role in TGF-β signaling. However, the function of proHp in cellular events downstream of Smads remains unclear. The current study investigated the effects of proHp on TGF-β-mediated Smad-dependent EMT induction and cell invasion *in vitro* using proHp-overexpressing SK-Hep1 liver cancer cells. The results of Western blotting, quantitative real-time RT-PCR, and immunocytochemistry indicated that proHp downregulated expression of mesenchymal marker and EMT regulator such as N-cadherin, vimentin, and twist, and upregulated expression of the epithelial marker E-cadherin. Compared with control cells, proHp-overexpressing cells exhibited high levels of ALK1/2/3 receptors and markedly increased Smad1/5 phosphorylation. Interestingly, proHp attenuated TGF-β-induced expression of mesenchymal markers and Smad2/3 phosphorylation. It also significantly suppressed cell invasion and migration. Knockdown of Smad1/5 abolished the inhibitory effects of proHp on TGF-β-stimulated Smad2/3 phosphorylation and mesenchymal marker expression. These findings indicate that proHp suppresses the TGF-β-induced EMT and cell invasion *in vitro* by enhancing Smad1/5 activation *via* ALK1/2/3 receptors and thus suppressing the Smad2/3 signaling pathway in SK-Hep1 cells. This study suggests that proHp may prevent a de-differentiation of hepatic cells and induce a cell differentiation by regulating the Smad signaling pathway.

## Introduction

Hepatocellular carcinoma (HCC) is one of the most common malignancies worldwide and has the fourth highest mortality rate [1]. Despite advances in diagnosis and treatment, the prognosis of HCC patients remains poor due to high rates of recurrence and metastasis.

The epithelial-mesenchymal transition (EMT) is a process of reversible de-differentiation in which epithelial cells acquire a mesenchymal phenotype [2, 3]. This process augments stem cell properties and prevents apoptosis and senescence. The EMT functions not only in embryonic development and tissue repair but also cancer progression by promoting cell migration and invasion [2]. During the EMT, epithelial cells lose cell-cell adhesion with repression of E-cadherin expression and acquire an invasive phenotype due to increased expression of mesenchymal marker and EMT regulator such as N-cadherin, vimentin, snail, and twist [2]. Transforming growth factor-β (TGF-β), Wnt, Notch, and tyrosine kinase receptor signals are part of the important signaling pathways associated with the EMT [4]. Cellular signals act cooperatively and promote the EMT during development and progression of hepatocarcinoma [5, 6].

TGF-β is considered a primary inducer of the EMT and its signaling is transduced *via* Smad-dependent and -independent pathways [7, 8]. In the canonical Smad pathway, the cytokine triggers the signal by binding to TGF-β receptor complexes consisting of type I and type II receptors with serine/threonine kinase activities. Binding of the ligand to type II receptors on the cell surface mediates recruitment and phosphorylation of type I receptors. Activated type I receptors phosphorylate downstream transcription factors called Smads. Of seven type I receptors referred to as activin receptor-like kinases (ALKs), ALK1, ALK2, ALK3, and ALK6 specifically phosphorylate Smad1/5/8, whereas ALK4, ALK5, and ALK7 are associated with Smad2/3 phosphorylation [9–11]. Phosphorylated Smad2/3 or Smad1/5/8 forms a complex with a common partner Smad4, which subsequently translocates into the nucleus and regulates expression of target genes [12, 13]. Activation of the Smad2/3 pathway plays a pivotal role in the TGF-β-induced EMT. The complex of Smad2/3 and Smad4 upregulates expression of central EMT regulators, members of the snail and twist families, which repress E-cadherin expression, a marker of epithelial cells [14, 15]. The role of Smad1/5/8 pathway in EMT induction is controversial. It was reported that Smad1/5 activation involves in EMT induction and cancer progression [16, 17], but another study showed that a negative regulator of TGF-β-mediated EMT increases Smad1/5/8 phosphorylation in cervical cancer cells [18]. It is necessary to clarify the function of Smad1/5/8 and the interactions of the Smad2/3 and Smad1/5/8 pathways during the TGF-β-induced EMT and cell invasion in liver cancer.

Haptoglobin (Hp), a scavenger of toxic hemoglobin, is a major acute phase protein [19, 20]. The plasma Hp level is increased in infectious diseases, cardiovascular diseases, and various types of cancer including HCC, pancreatic cancer, colon cancer, and lung cancer [19, 20]. The liver is a main site of Hp biosynthesis. In hepatocytes, the *Hp* gene is expressed in a precursor form, prohaptoglobin (proHp), in which the α- and β-subunit are linked within a single polypeptide [21, 22]. ProHp is then site-specifically cleaved at the junction between the α- and β-subunit by the C1r-like protein (C1r-LP), which contains the serine protease domain of complement C1r. The separated α- and β-chains are held together by disulfide bonds and assembled into the mature Hp form. In the endoplasmic reticulum, proHp is processed to the mature form Hp and secreted into the circulation [23]. We recently found that proHp stimulates endothelial angiogenesis *in vitro* and detected it in sera from liver cancer patients [24]. Moreover, we previously demonstrated that the angiogenic effect of proHp is dependent on Smad1/5 activation *via* ALK1 in endothelial cells [25], suggesting that proHp plays a role in TGF-β signaling. However, the functions of proHp in cellular events downstream of Smads remain unknown. In the current study, we investigated its effects on TGF-β-mediated Smad-dependent EMT induction and cell invasion using proHp-overexpressing SK-Hep1 liver cancer cells. The interaction between Smad2/3 and Smad1/5 signaling was also explored in proHp-overexpressing cells. Interestingly, we found that proHp inhibits the TGF-β-induced EMT.

## Materials and methods

### Cell culture and transient transfection with *Hp* gene

The SK-Hep1 human liver cancer cell line was purchased from the Korean Cell Line Bank (KCLB, Seoul, Korea). The cells were cultured in Dulbecco’s Modified Eagle Medium (DMEM; WelGENE, Daegu, Korea) supplemented with 10% fetal bovine serum (FBS; Gibco/ThermoFisher Scientific, Waltham, MA, USA) and maintained at 37°C in a humidified incubator with 5% CO_2_. To generate proHp-overexpressing cells, SK-Hep1 cells were transfected with *Hp2* cDNA [26] subcloned into the EcoRI and KpnI sites of the pcDNA3.0 expression vector (Invitrogen, Carlsbad, CA, USA) using Lipofectamine 2000 (ThermoFisher Scientific), according to the manufacturer’s protocol. The cells transfected with vehicle pcDNA3.0 vector were used as a control group. After the transiently transfected SK-Hep1 cells were cultured for 48 h in serum-free DMEM, Hp protein secreted into the culture medium (CM) was identified by Western blot analysis. In the experiment to examine the effect of mature form Hp, SK-Hep1 cells were treated with Hp2-2 purified from normal human serum (SRP6508, Sigma-Aldrich, St. Louis, MO, USA) for 48 h in DMEM containing 10% FBS.

### Western blotting

The CM (30μl) of *Hp2*-transfected SK-Hep1 cells was electrophoresed on a 10% SDS-polyacrylamide gel under reducing conditions and the separated proteins were transferred to a nitrocellulose membrane. Immunoreactions were performed with a rabbit polyclonal anti-human Hp antibody (H8636, Sigma-Aldrich) and a polyclonal anti-human Hp α-chain antibody, which was produced in our laboratory [27].

To detect EMT-related proteins, SK-Hep1 cells were transfected with *Hp2* cDNA and then incubated for 24 h in DMEM containing 10% FBS. After incubation, the cells were treated with TGF-β (5 ng/mL) for 48 h (E-cadherin and vimentin) or 1 h (Smad phosphorylation). In experiments to analyze Smad phosphorylation, the cells were preincubated for 3 h in serum-free medium before TGF-β treatment. The cell lysates were analyzed with a monoclonal antibody against E-cadherin (ab40772, Abcam, Cambridge, UK) or vimentin (ab92547, Abcam). For detection of Smads, the monoclonal antibodies were obtained from Cell Signaling Technologies (Danvers, MA, USA); anti-Smad2/3 (8685S), anti-Smad1 (6944S), anti-phospho-Smad2/3 (8828S), and anti-phospho-Smad1/5antibodies (9516S). Immunoreactive signals were detected using an enhanced chemiluminescence assay kit (Amersham Biosciences, Buckinghamshire, UK) and a luminescence image analyzer (LAS-3000; Fujifilm, Tokyo, Japan).

### Reverse-transcription PCR (RT-PCR) and quantitative real-time RT-PCR

Total RNA was isolated using TRIzol (ThermoFisher Scientific), and 2 μg of total RNA was reverse-transcribed into cDNA for 1 h at 42°C using M-MLV reverse transcriptase (Promega, Madison, WI, USA). The cDNA was amplified on a MyCycler Thermal Cycler (Bio-Rad, Hercules, CA, USA) using GoTaq DNA polymerase (Promega). The primers used in this study are shown in Table 1. Each PCR cycle comprised 94°C for 1 min, 60°C for 1 min, and 72°C for 1 min. The PCR products were electrophoresed on a 1% agarose gel containing 0.01% (v/v) SafeView nucleic acid dye (abm Inc., Richmond, BC, Canada) and photographed using the Gel Doc XR+ System (Bio-Rad).

**Table 1:** Oligonucleotide primers used in this study.

Gene	Primer sequence	Size (bp)
E-cadherin	5′-GCCTCCTGAAAAGAGAGTGGAAG-3′	131
	5′-TGGCAGTGTCTCTCCAAATCCG-3′	
N-cadherin	5′-CCTCCAGAGTTTACTGCCATGAC-3′	149
	5′-GTAGGATCTCCGCCACTGATTC-3′	
Vimentin	5′-GAGAACTTTGCCGTTGAAGC-3′	163
	5′-GCTTCCTGTAGGTGGCAATC-3′	
Twist	5′-GGAGTCCGCAGTCTTACGAG -3′	201
	5′-TCTGGAGGACCTGGTAGAGG-3′	
ALK-1	5′-GGCTCCCTCTACGACTTTCT -3′	134
	5′-TGGGCAATGGCTGGTTT -3′	
ALK-2	5′-CAAAATCCATCCGCAAGACT-3′	143
	5′-GCTGGACAATGACAACAACG -3′.	
ALK-3	5′-AGCCTCCAGACTCACAGCAT-3′	134
	5′-CATGCCATGGGTAAAAACAG-3′	
ALK-4	5′-GCGTGTCTATCACAACCG -3′	345
	5′-TCAGCAGCAATAAATCCAA -3′.	
ALK-5	5′-ATCCCAAACAGATGGCAGAG -3′	190
	5′-GGAGAGTTCAGGCAAAGCTG -3′.	
ALK-6	5′-CCTGCGGGTTAAGAAAACAC-3′	122
	5′-CTCTGCCCACAAACAGAAGA-3′.	
GAPDH	5′-GTCTCCTCTGACTTCAACAGCG-3′	131
	5′-ACCACCCTGTTGCTGTAGCCAA-3′.	

Quantitative real-time PCR was performed on a MX300P thermal cycler (Stratagene, San Diego, CA, USA) using SYBR Green Master Mix (Applied Biosystems, Foster City, CA, USA) according to the manufacturer’s instructions. Gene expression levels were normalized to that of GAPDH.

### Fluorescence immunocytochemistry

Cells were fixed with 4% formaldehyde (Wako, Osaka, Japan), permeabilized with 0.1% Triton X-100, and blocked with 1% bovine serum albumin prepared in phosphate-buffered saline (PBS). Cells were incubated with an anti-human E-cadherin (ab40772, Abcam) or vimentin antibody (ab92547, Abcam) at 4°C overnight and then with donkey anti-rabbit IgG (ab150073, Alexa Fluor® 488, abm Inc.) or goat anti-rabbit IgG (# A-11011, Alexa Fluor® 568, Thermo Fisher) for 2 h. Finally, cells were washed and mounted with mounting medium containing DAPI (Abcam). Images were acquired using a confocal microscope (LSM800; Carl Zeiss, Inc., Jena, Germany).

### Cell invasion and wound healing assays

Cell invasion assays were performed in 24-well transwell plates with polycarbonate filters with a pore size of 8.0 μm (Costar, Corning, NY, USA). Transwell insert membranes were precoated with 1 mg/mL matrigel (BD Biosciences, San Jose, CA, USA) for 2 h. Cells (5 × 10^4^) in serum-free DMEM containing or lacking 5 ng/mL TGF-β1 (R&D Systems, Minneapolis, MN, USA) were seeded in the upper chamber and 10% FBS-containing DMEM was added to the lower chamber. After incubation for 24 h, non-migrating cells on the upper surface of the insert were removed with a cotton swab, and invading cells on the lower surface of the membrane were fixed in 100% methanol for 20 min and stained with 1% crystal violet. Invading cells were photographed under an inverted microscope and counted in three randomly selected fields per group.

For wound healing assays, cell monolayers were scratched with a 1 mL pipette tip and washed with PBS to remove unattached cells. Cells were then cultured in the absence or presence of 5 ng/mL TGF-β1 in DMEM containing 1% FBS for 24 h. Cell migration was observed and images were captured under a microscope. Cell migration was quantified by measurement at 14 random sites using ImageJ software (National Institutes of Health).

### Knockdown of Smad1/5

To silence expression of Smad1/5, specific siRNAs targeting Smad1 and Smad5 (ON-TARGETplus SMARTpool) and a negative control siRNA (siCONTROL Non-Targeting siRNA Pool) were purchased from Dharmacon (Chicago, IL, USA). The siRNAs were introduced into the proHp-overexpressing SK-Hep1 cells according to the manufacturer’s protocol. Briefly, 25 nM (final concentration) of a mixture of Smad1- and Smad5-targeting siRNAs or a negative control siRNA was mixed with DharmaFECT4 transfection reagent and incubated for 20 min at room temperature. The transfection mixture was applied to the SK-Hep1 cells which were previously transfected with *Hp2* gene for 24 h. After the cells were further incubated for 24 h, the cells were treated with TGF-β (5ng/mL) for 48 h (E-cadherin and vimentin) or 1 h (Smad phosphorylation). In experiments to analyze Smad phosphorylation, the cells were preincubated for 3 h in serum-free medium before TGF-β treatment.

### Statistical analysis

The results are expressed as the mean ± standard deviation (SD). Significance was determined using the Student’s t-test or a one-way analysis of variance followed by Tukey’s multiple comparison test. All statistical analyses were performed using GraphPad Prism v8.0.1 (GraphPad Software, San Diego, CA, USA). A p-value less than 0.05 was considered statistically significant.

## Results

### *Hp* gene-transfected SK-Hep1 cells produce proHp

**Fig 1:**
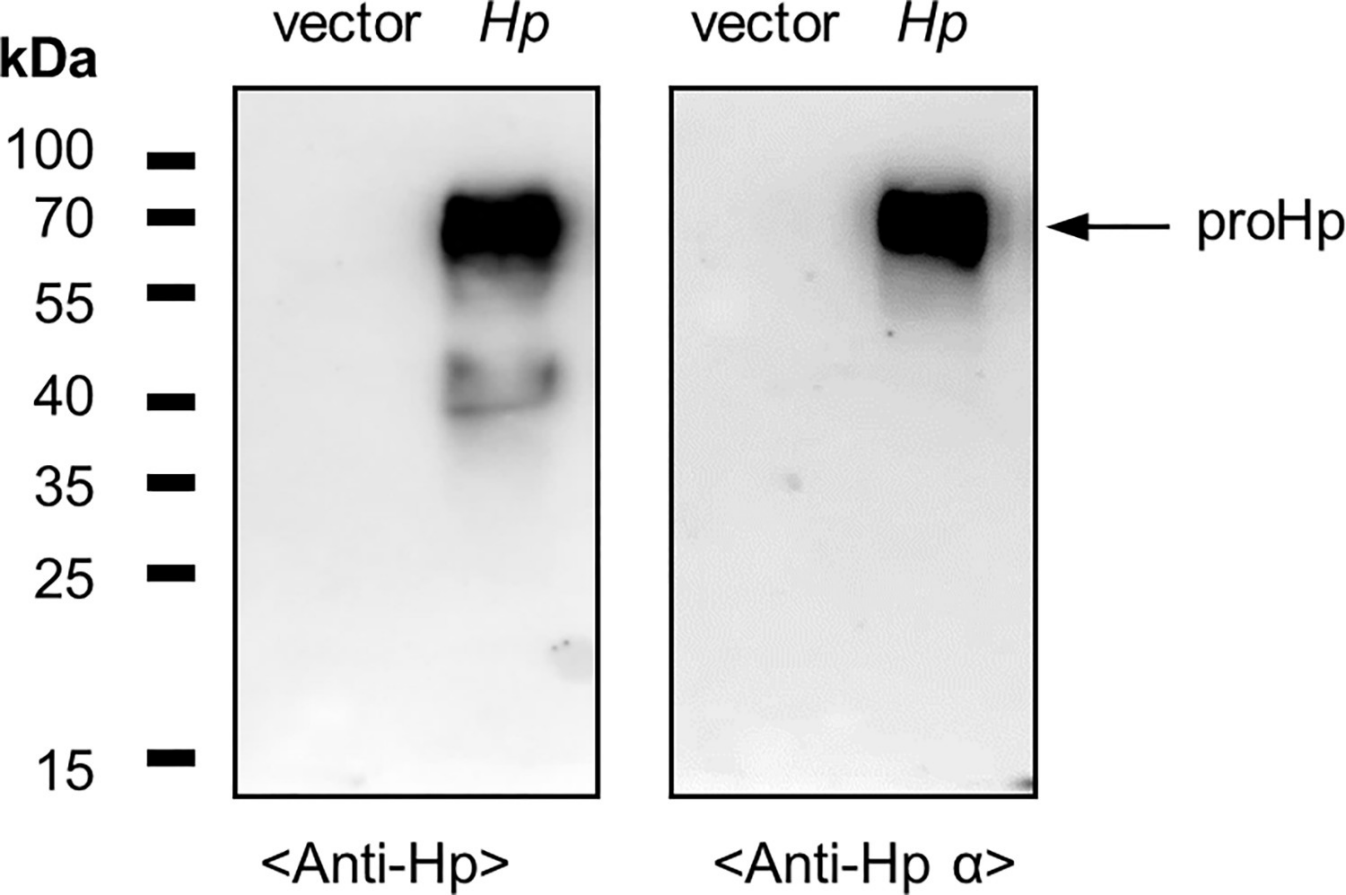
Identification of proHp in the CM of *Hp* gene-transfected SK-Hep1 cells. SK-Hep1 cells were transfected with vehicle vector DNA (vector) or vector containing human *Hp2* cDNA (*Hp*) using Lipofectamine 2000. After cells had been cultured for 48 h in serum-free DMEM, Hp protein released into CM was detected by Western blotting using anti-Hp and anti-Hp α-chain antibodies. Note that the produced protein is the precursor proHp.

The hepatic Hp expression is dependent on liver development and cell differentiation. Hp is actively synthesized in well-differentiated hepatocytes, but not expressed in undifferentiated stem cells [28, 29]. SK-Hep1 cells are poorly differentiated hepatoma cells with characteristics of mesenchymal stem cells [30, 31]. Although SK-Hep1 cell line has been widely used as a cell model of hepatocarcinoma, these cells do not produce a hepatic protein albumin [32]. As expected, Hp/proHp was not detected in control SK-Hep1 cells (Fig 1). To investigate the potential role of Hp/proHp on the EMT and invasion of hepatoma cells, the human *Hp2* gene was introduced into SK-Hep1 cells. The *Hp2*-transfected SK-Hep1 cells produced the protein as the precursor proHp containing a single polypeptide with a size of 68 kDa (Fig 1), rather than as the mature form Hp containing the separated α- and β-chains. Therefore, *Hp2*-transfected SK-Hep-1 cells were used to study the functions of proHp in hepatoma cells.

### proHp overexpression attenuates mesenchymal properties and invasion of SK-Hep1 cells

**Fig 2:**
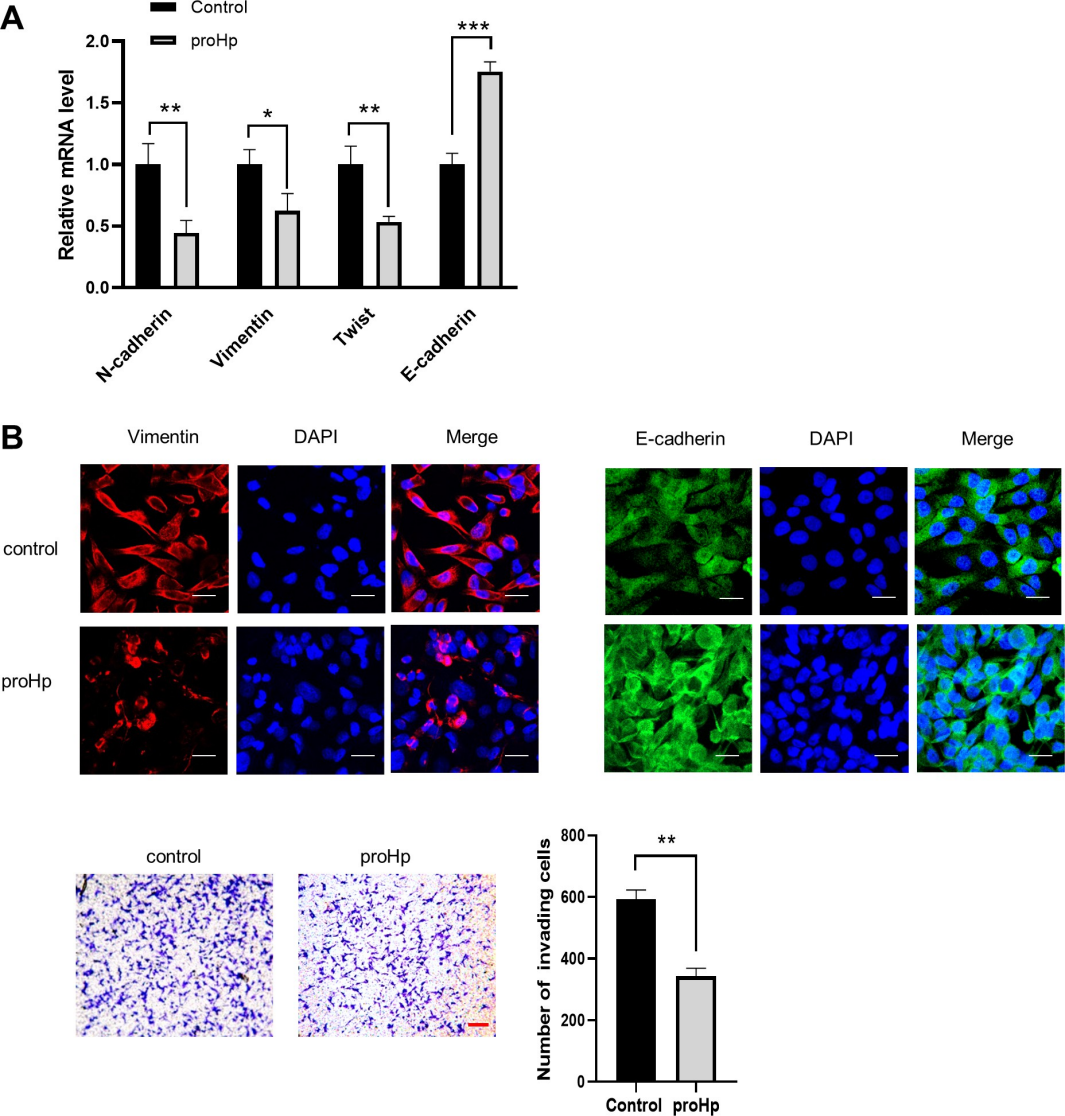
proHp overexpression reduces mesenchymal properties and invasion of SK-Hep1 cells. proHp-overexpressing cells transfected with the *Hp* gene and control cells transfected with vehicle vector DNA were cultured for 48 h in DMEM containing 10% FBS. (A) mRNA levels of N-cadherin, vimentin, twist, and E-cadherin were determined by qRT-PCR. The expression levels were normalized to GAPDH. Data are presented as the mean ± SD of triplicate experiments. *P<0.05, **P<0.01, ***P<0.001 vs. vector-transfected control cells (Student’s t-test). (B) Immunofluorescence staining of vimentin and E-cadherin in control and proHp-overexpressing cells was detected by confocal microscopy (scale bar = 25 μm). Results are representative of independent experiments repeated twice. (C) Cell invasion was evaluated by a transwell assay. Cells in serum-free medium were added to the upper chamber and 10% FBS-containing DMEM was added to the lower chamber. After incubation for 24 h, invading cells were stained, photographed under an inverted microscope (scale bar = 100 μm), and counted in three randomly selected fields per group. Results are representative of three independently performed experiments. **P<0.01 vs. vector-transfected control cells (Student’s t-test).

We first evaluated expression of EMT markers using real-time qRT-PCR and immunocytochemistry. mRNA levels of the N-cadherin, vimentin, and twist were reduced in proHp-overexpressing SK-Hep1 cells (Fig 2A). On the contrary, expression of the epithelial cell marker E-cadherin was higher in proHp-overexpressing cells than in vector-transfected control cells (Fig 2A). The protein levels of vimentin and E-cadherin were also decreased and increased, respectively, in proHp-overexpressing cells (Fig 2B). In addition, a transwell invasion assay demonstrated that proHp overexpression inhibited invasion of SK-Hep1 cells up to 1.73-fold (Fig 2C). These findings indicate that proHp attenuates mesenchymal properties and invasion of hepatoma cells.

### proHp prevents the TGF-β-induced EMT and cell invasion

**Fig 3:**
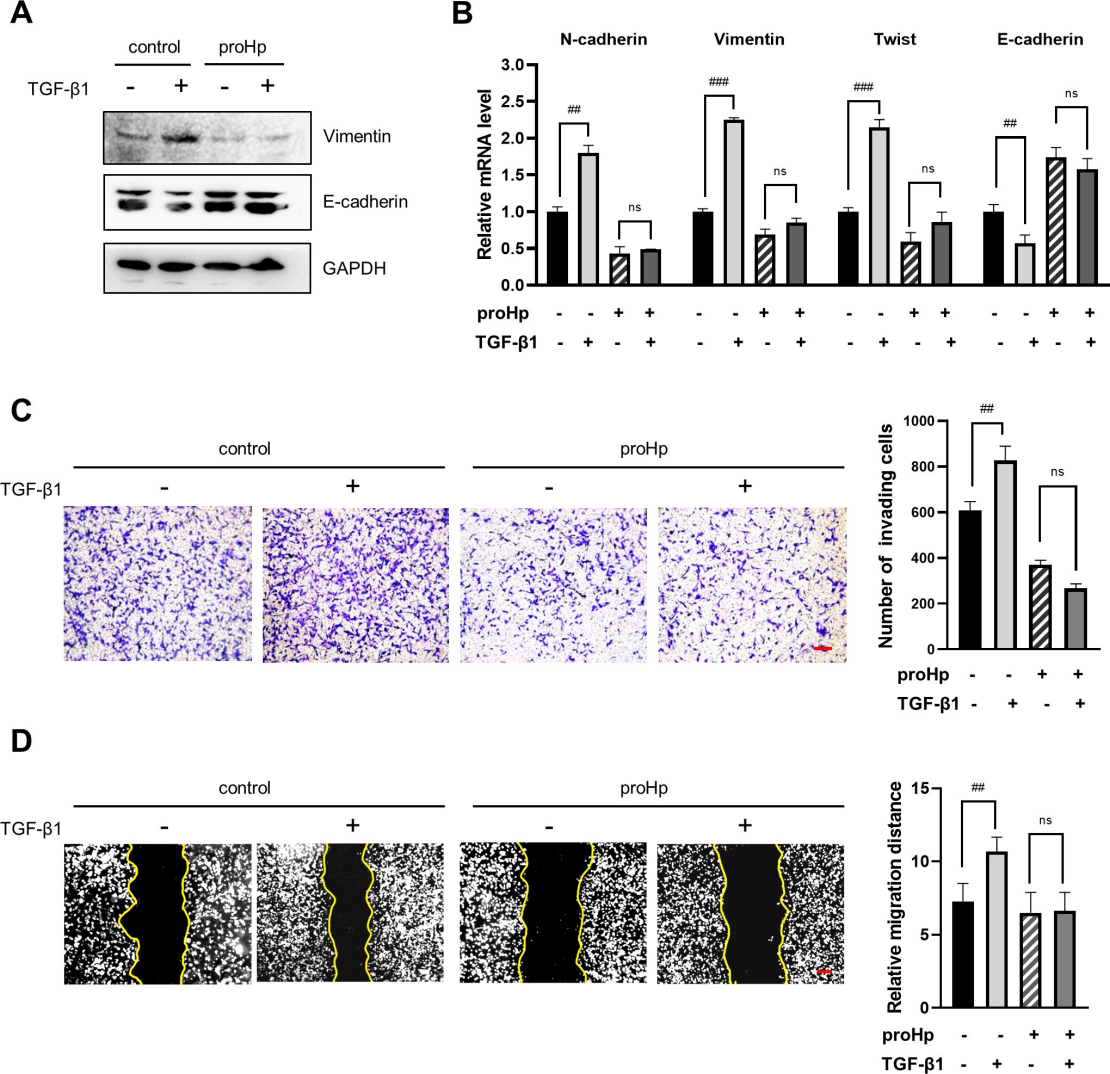
proHp attenuates the TGF-β-induced EMT. Control and proHp-overexpressing cells were treated with 5 ng/mL TGF-β for 48 h. (A) Expression of vimentin and E‑cadherin in cell lysates was detected by Western blotting. (B) The mRNA levels of N-cadherin, vimentin, twist, and E-cadherin were determined by qRT-PCR and shown as relative levels. (C) Cells in serum-free media containing or lacking 5 ng/mL TGF-β were seeded in the upper chamber of transwell inserts and 10% FBS-containing DMEM was added to the lower chamber. After incubation for 24 h, invading cells were photographed under a microscope (scale bar = 100 μm) and counted in three randomly selected fields per group. (D) Wound healing assays were performed in medium lacking or containing 5 ng/mL TGF-β. After culture for 24 h, cell migration was observed under a microscope and the migration distances were quantified at 14 random sites in the captured images using ImageJ software. All quantitative results are presented as the mean ± SD of triplicate experiments. ^##^*P*<0.01, ^###^*P*<0.001 vs. no TGF-β treatment. “ns” means not significant (*P*>0.05 vs. TGF-β-untreated proHp-overexpressing cells, Student’s t-test). All figures are representative of independent experiments performed at least twice.

TGF-β is a major inducer of the EMT in various cancer cells. Therefore, we next investigated whether proHp diminishes the TGF-β-stimulated EMT and cell invasion. When control SK-Hep1 cells lacking proHp were treated with 5 ng/mL TGF-β for 48 h, expression of mesenchymal marker and EMT regulator (N-cadherin, vimentin, and twist) obviously increased, while expression of E-cadherin decreased (Fig 3A and 3B). However, TGF-β little affected on the expression of EMT markers in proHp-overexpressing cells (Fig 3A and 3B). Moreover, proHp significantly blocked the stimulatory effects of TGF-β on cell invasion and migration (Fig 3C and 3D). These results indicate that proHp inhibits the TGF-β-induced EMT in SK-Hep1 cells.

### proHp inhibits TGF-β-stimulated Smad2/3 phosphorylation

**Fig 4:**
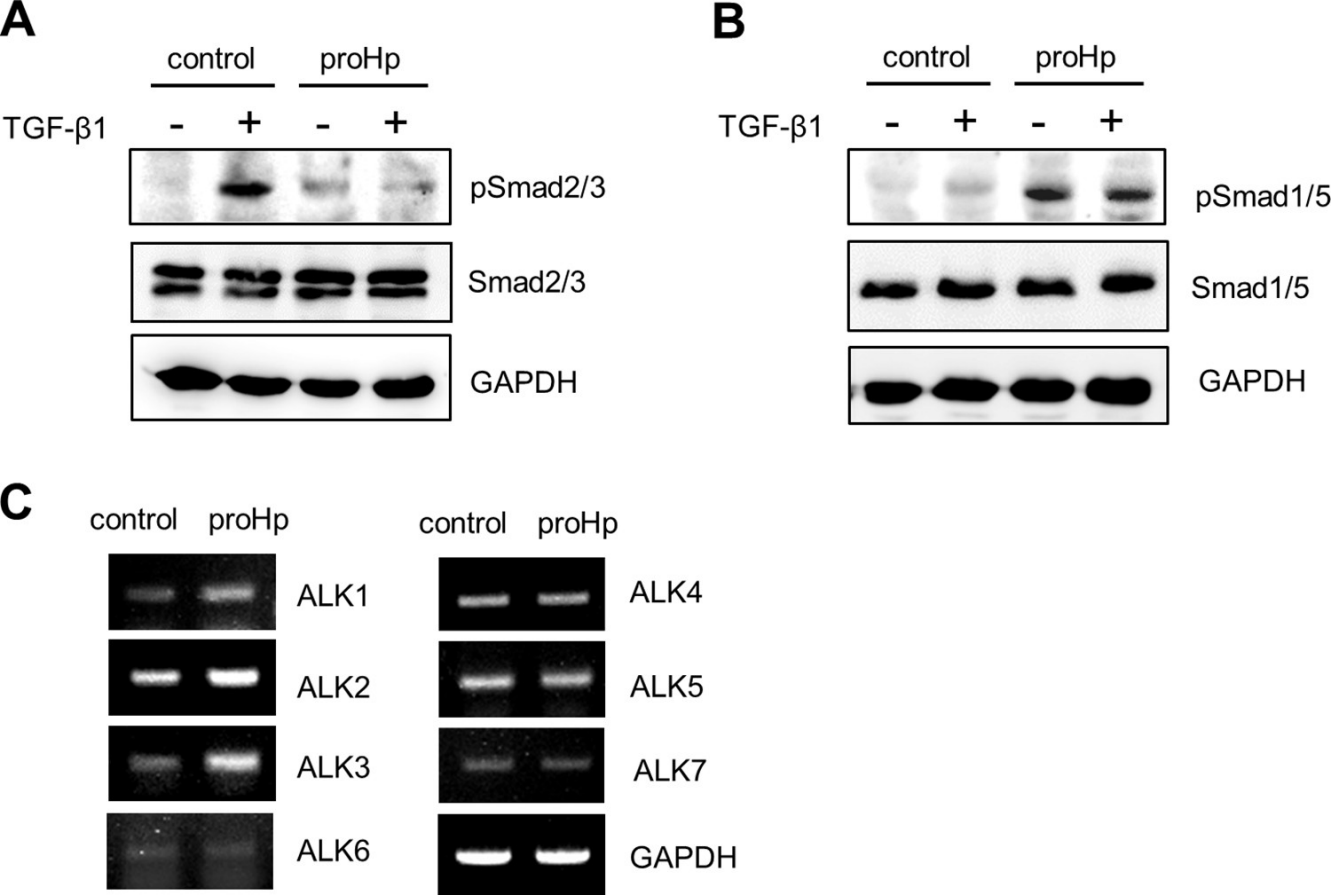
proHp inhibits TGF-β-induced Smad2/3 phosphorylation and increases expression of ALK1/2/3 receptors in SK-Hep1 cells. After transfection with vector DNA (control) or *Hp* gene (proHp), the cells were incubated for 24 h in DMEM containing 10% FBS. (A and B) For serum starvation, the cells were further incubated for 3 h in serum-free medium, and then 5ng/mL TGF-β was added into the culture medium. After treatment for 1 h, phosphorylation of Smad2/3 (pSmad2/3) and Smad1/5 (pSmad1/5) in cell lysates was detected by Western blot analysis. (C) mRNA levels of TGF-β type I receptors (ALK1 to ALK7) in control and proHp-overexpressing cells were determined by RT-PCR. All experiments were performed at least twice with similar results, and representative data are shown.

To investigate the mechanism by which proHp attenuates the EMT, we examined TGF-β-triggered Smad2/3 phosphorylation in cells containing or lacking proHp. Treatment with 5 ng/mL TGF-β for 1 h greatly increased phosphorylation of Smad2/3 in control SK-Hep1 cells, but did not induce Smad2/3 phosphorylation in proHp-overexpressing cells (Fig 4A). This implies that proHp inhibits the TGF-β-stimulated Smad2/3 signaling pathway.

Our previous study demonstrated a novel function of proHp in Smad1/5 activation in endothelial cells [25]; therefore, we investigated the effect of proHp on the Smad1/5 pathway during the EMT in hepatoma cells. Treatment with 5 ng/mL TGF-β for 1 h slightly enhanced Smad1/5 phosphorylation in vehicle vector-transfected control cells (Fig 4B). However, phosphorylation of Smad1/5 was highly augmented in both non-treated and TGF-β-treated proHp-overexpressing cells (Fig 4B). To investigate whether the distinct effects of proHp on the Smad phosphorylations are owing to differences in expression of TGF-β receptors, the levels of TGF-β type I receptors (ALK1 to ALK7) were determined by RT-PCR. Expression of ALK1, ALK2, and ALK3, which are related to the Smad1/5/8 activation, was increased in proHp-overexpressing cells, but expression of ALK4, ALK5, and ALK7, which are related to Smad2/3 signaling, was not affected (Fig 4C). These findings suggest that proHp enhances expressions of the ALK1/2/3 TGF-β type I receptors and this leads to activation of the Smad1/5/8 signaling pathway.

### Knockdown of Smad1/5 abolishes the inhibitory effects of proHp in TGF-β-activated Smad2/3 pathway

**Fig 5:**
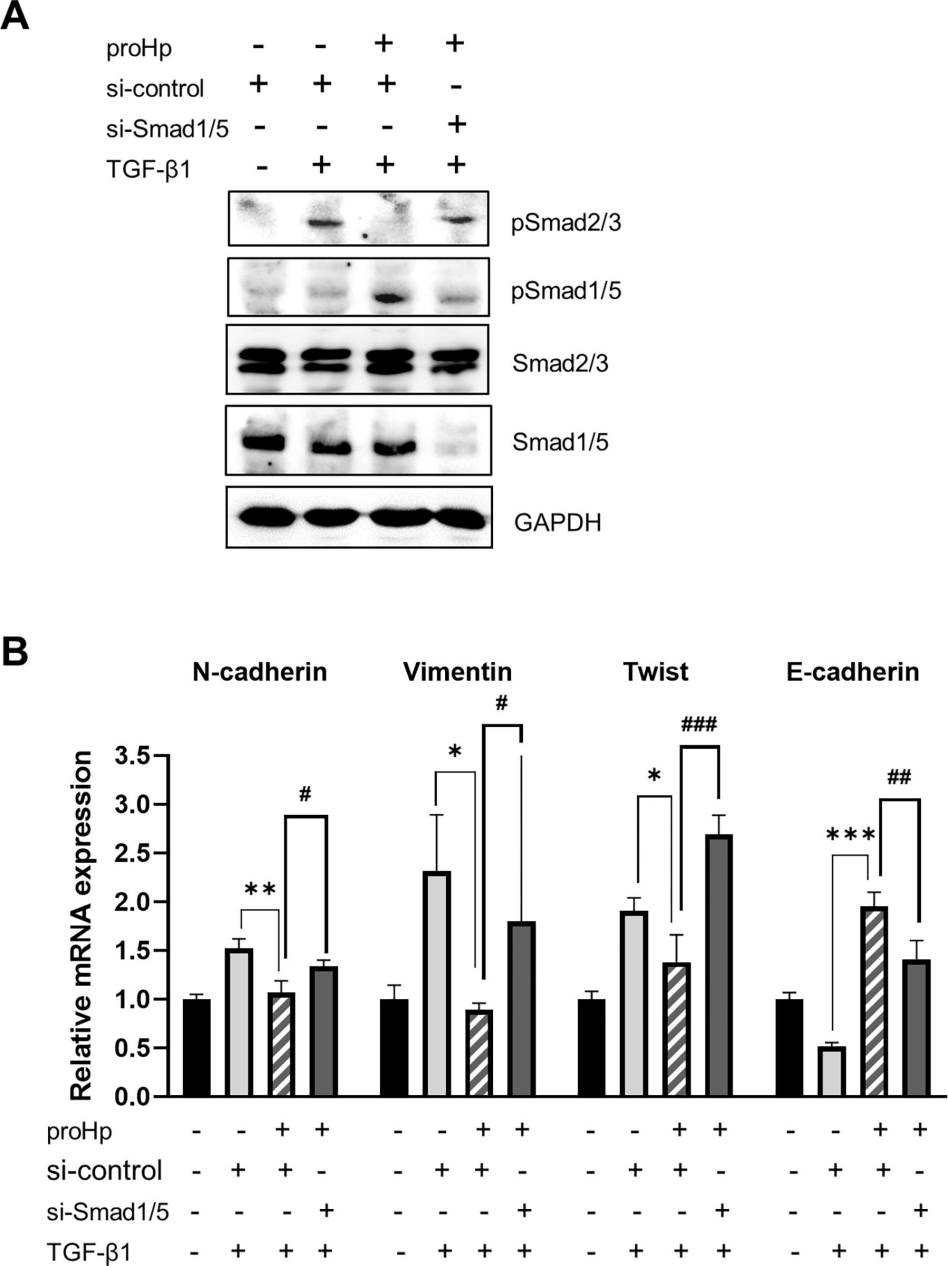
Smad1/5 knockdown recovers TGF-β-induced Smad2/3 activation in proHp-overexpressing cells. ProHp-overexpressing SK-Hep1 cells were transfected with control (si-control) or Smad1/5-targeting (si-Smad1/5) siRNA as described in the Materials and methods. (A) After serum-starvation for 3 h, the cells were stimulated with 5 ng/mL TGF-β for 1 h. Phosphorylation of Smad2/3 and Smad1/5 was analyzed by Western blotting. (B) After incubation of the gene-knockdown cells with 5 ng/mL TGF-β for 48 h, mRNA expression of N-cadherin, vimentin, twist, and E-cadherin was quantified by qRT-PCR. Expression levels were normalized to GAPDH. Data are presented as the mean ± SD of triplicate experiments. *P<0.05, **P<0.01, ***P<0.001 vs. vector-transfected control cells. ^#^*P*<0.05, ^##^*P*<0.01, ^###^*P*<0.001 vs. control siRNA-transfected cells (one-way ANOVA). All experiments were performed at least twice with similar results, and representative data are shown.

To determine whether proHp-activated Smad1/5 directly affects Smad2/3-dependent TGF-β signaling, Smad1/5 was knocked down. proHp-overexpressing cells were transfected with Smad1/5-targeting siRNAs, stimulated with 5 ng/mL TGF-β, and analyzed by Western blotting and qRT-PCR. The introduction of Smad1/5 siRNA into the cells blocked Smad1/5 expression and down-regulated the phosphorylation of Smad1/5, but did not affect Smad2/3 expression (Fig 5A). In control siRNA-transfected SK-Hep1 cells, TGF-β treatment increased Smad2/3 phosphorylation but the TGF-β-induced Smad2/3 phosphorylation was markedly attenuated by proHp overexpression. However, Smad1/5-silencing in proHp-overexpressing cells did not reduce the Smad2/3 phosphorylation (Fig 5A). Moreover, Smad1/5 knockdown abolished the inhibitory effects of proHp on expression of N-cadherin, vimentin, and twist, and its stimulatory effect on expression of E-cadherin (Fig 5B). These results indicate that proHp-stimulated Smad1/5 activation is directly related to inhibition of the Smad2/3 signaling pathway, suggesting that proHp participates in regulatory interactions between these two Smad signaling pathways in SK-Hep1 hepatoma cells.

## Discussion

We transfected SK-Hep1 liver cancer cells with human *Hp* cDNA and investigated the effects on TGF-β-triggered Smad-dependent EMT induction and cell invasion *in vitro*. *Hp* gene transfected into the SK-Hep1 cells was expressed mostly as the unprocessed precursor from proHp rather than as the mature from Hp (Fig 1). Sometimes, besides the proHp, a little mature Hp containing the separated α- and β-chains was detected in theses transfected cells, However, amount of the mature Hp was much less than that of the proHp. Therefore, we considered the results of this study to indicate the functions of proHp. The Hp-processing enzyme C1r-like protein has been identified in well-differentiated hepatocytes [23]. Perhaps, the processing enzyme is likely deficient in the SK-Hep1 cells which have a poorly differentiated stem-like property. Until now, we do not know whether mature Hp affects the inhibition of the EMT in liver cancer. In preliminary test, when 0.1 mg/mL Hp2-2 (purified from normal human serum, Sigma) was exogenously added to the SK-Hep1 culture medium and incubated for 48 h, the expression of vimentin and E cadherin was decreased and increased, respectively (S1 Fig). It suggests that mature Hp may also reduce a mesenchymal property of liver cancer cells. However, to compare the function of mature Hp with that of proHp on the EMT inhibition, an elaborative study will be required to use the same conditioned materials such as recombinant proHp/Hp or control SK-Hep1 cells/C1r-like protein-overexpressing cells.

SK-Hep1 cell line was derived from the ascitic fluid of a patient with adenocarcinoma of the liver, and the cells can form large cell carcinoma consistent with hepatoma in nude mice (The American Type Culture Collection). It has been also reported that SK-Hep1 is a poorly-differentiated hepatoma cell line and exhibits characteristics of mesenchymal stem cells with a high metastatic activity [30, 31]. Although SK-Hep1 cells have been widely used as a cell model of HCC, the cell line was identified as being of endothelial origin [32]. In the current study, we used the proHp-overexpressing SK-Hep1 cell model and demonstrated the suppressing effects of proHp on the EMT induction and cell invasion. However, it is unclear whether this activity of proHp is limited to unusual specific hepatoma cells such as SK-Hep1 or can be applicable to wide spectrum of hepatoma. It is necessary to confirm this proHp function in various hepatocarcinoma cells.

In human, the Hp protein exists as various proteoforms owing to gene polymorphisms, processing during biosynthesis, and post-translational modifications. Three major phenotypes according to gene polymorphisms (Hp1-1, Hp2-1, and Hp2-2), unprocessed precursors (proHp1 and proHp2), and unusually glycosylated Hp (especially fucosylated Hp) have been reported and they play diverse roles in inflammation, diabetic vascular disease, angiogenesis, and malignant tumors [19, 20, 24]. The plasma Hp level, Hp expression in tumor cells, and Hp fucosylation are reportedly related to cancer development and have been suggested to be candidate biomarkers of various cancers including HCC, colorectal cancer, pancreatic cancer, and glioblastoma [20, 33, 34]. In addition, a preliminary report showed an association of tissue Hp expression and the EMT in buccal cancer [35]. On the contrary, an interesting recent study demonstrated that Hp expression in tumors positively correlated with differentiation of HCC and the 5-year overall survival rate of patients [36]. These conflicting findings concerning the function of Hp in development and differentiation of HCC may be owing to diverse Hp proteoforms expressed in malignant tumor tissues as well as cancer types. Therefore, the Hp isoforms must be carefully identified in Hp functional studies.

While there are many studies of Hp, the distinct function of proHp remains unclear. A few studies have described proHp as a human zonulin (modulator of cell tight junction permeability) and an activator of B cell survival and differentiation [37, 38]. We previously reported that proHp stimulates endothelial angiogenesis and that the angiogenic function of proHp is associated with TGF-β signaling *via* ALK1-Smad1/5 activation [24, 25]. The current study indicates that proHp blocks TGF-β-stimulated Smad2/3 activation and attenuates mesenchymal properties and invasion of SK-Hep1 cells (Figs 3 and 4). Our results imply that proHp can reverse the EMT, showing the downregulation of EMT markers (N-cadherin, vimentin, and twist) and the upregulation of epithelial marker (E-cadherin). Moreover, proHp inhibited cell invasion and migration. These findings suggest that proHp may contribute to differentiation of hepatoma cells and suppression of HCC progression. However, it is still unclear whether proHp exits in tumor tissues or microenvironment of hepatoma cancers, while the presence of proHp in the sera of patients with hepatic and pancreatic cancers has been shown [24, 39]. A recent study demonstrated that immune cells, particularly macrophages, in pancreatic cancer microenvironment produce fucosylated proHp [40]. If *in vivo* evidences are accumulated for the proHp expression and its regulating functions on EMT in liver cancer microenvironment, proHp could be proposed as a candidate biomarker of HCC metastasis and progression.

**Fig 6:**
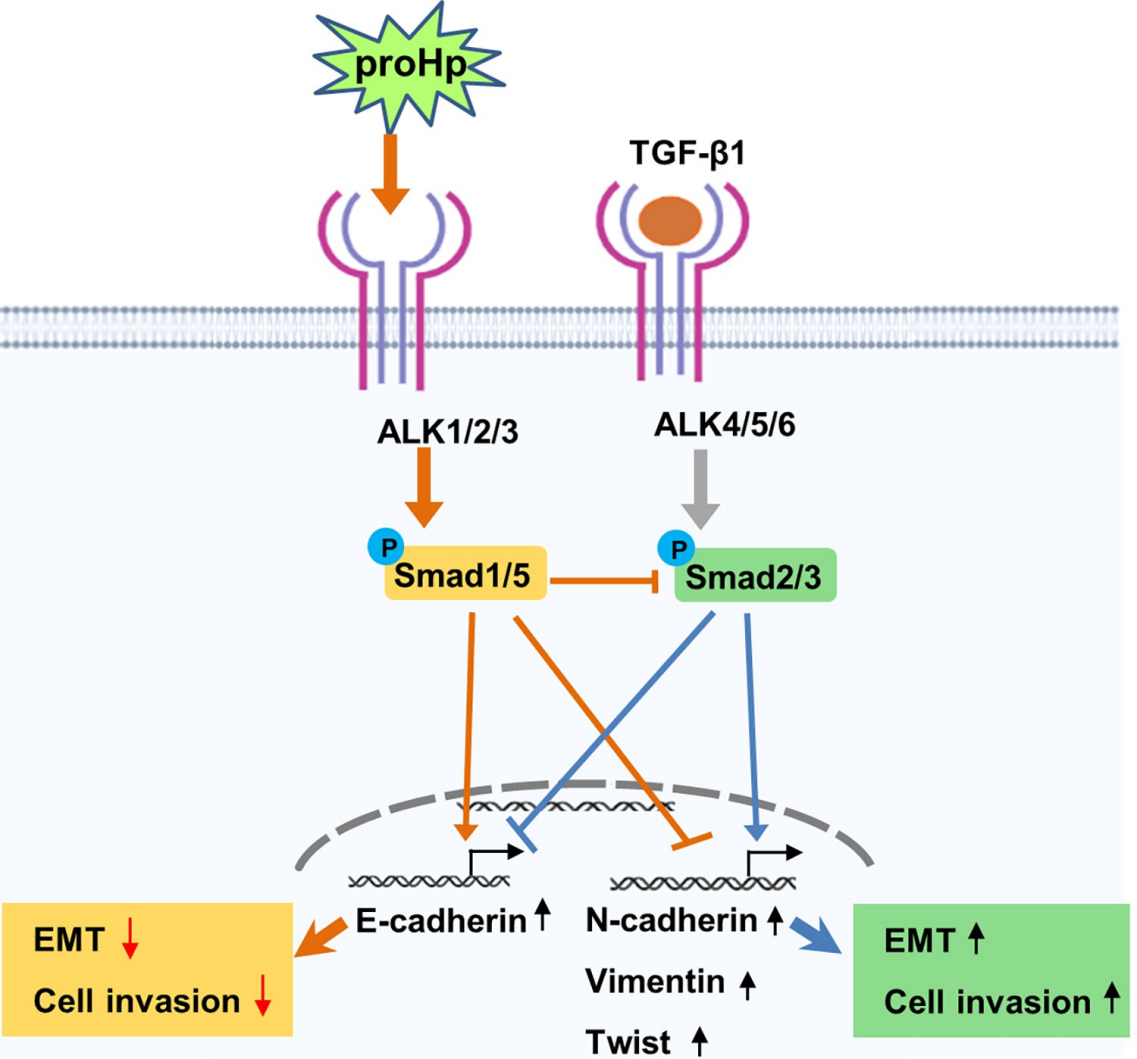
The proposed mechanism by which proHp inhibits the TGF-β-induced EMT in SK-Hep1 liver cancer cells.

TGF-β is a key inducer of the EMT, which is an important step in cancer progression, and Smad2/3 activation plays a crucial role in the EMT. Our observations revealed that proHp inhibits the EMT and cell invasion by preventing stimulation of Smad2/3 phosphorylation in SK-Hep1 cells. Overexpression of proHp in this cell line markedly upregulated Smad1/5 phosphorylation and expression of ALK1/2/3 receptors (Fig 4). Silencing of Smad1/5 restored TGF-β-induced Smad2/3 phosphorylation and mesenchymal properties (Fig 5); therefore, proHp-induced Smad1/5 activation is thought to directly suppress the Smad2/3 signaling pathway. Based on the findings of this study, we propose a mechanism underlying the novel function of proHp. Specifically, proHp upregulates Smad1/5 phosphorylation *via* ALK1/2/3 receptors and thereby disturbs the TGF-β-stimulated Smad2/3 signaling pathway. Consequently, expression of N-cadherin, vimentin, and twist is reduced, whereas that of E-cadherin is increased. Owing to the proHp-induced reduction of mesenchymal properties, cell invasion is also inhibited (Fig 6).

Our results are consistent with the findings of Goto *et al*. [18]. They demonstrated that FAD104, a positive regulator of adipocyte differentiation, suppresses the TGF-β-mediated EMT in cervical cancer cells, with upregulation of Smad1/5/8 phosphorylation and downregulation of Smad2/3 phosphorylation. Moreover, Goumans *et al*. suggested that activation of Smad1/5 signaling *via* the ALK1 receptor directly antagonizes the Smad2/3 pathway *via* TGF-β/ALK5 in endothelial cells [41]. However, a molecular mechanism by which SMAD1/5/8 activity would downregulate SMAD2/3 signals still remains to be addressed. As a potential mechanism, it could be thought the competition between receptors in ligand binding. In the conditions that ALK1/2/3 receptors associated with SMAD1/5/8 activation are highly expressed, the superior binding of ligand to these receptors can interrupt the SMAD2/3 signaling pathway. In addition, it is needed to examine the expression of co-receptors/accessory receptors such as endoglin, which potentiates TGF-β/ALK1 signaling and negatively acts on TGF-β/ALK5 signaling [42]. Sequestering of co-SMAD4 by SMAD1/5 could be also involved in the mechanism. A detailed further study is required to verify the interaction and crosstalk between the two TGF-β signaling pathways through Smad2/3 activation and Smad1/5/8 activation during EMT induction and metastasis of hepatoma cells.

In summary, we demonstrated for the first time that proHp suppresses the TGF-β-induced EMT and cell invasion *in vitro* by enhancing Smad1/5 activation and suppressing the Smad2/3 signaling pathway in SK-Hep1 liver cancer cells. Our results suggest that proHp is a candidate biomarker to identify the degree of differentiation and metastatic ability of HCC cells. However, this study has a limitation of no *in vivo* evidences to support the inhibitory role of proHp on cancer metastasis. For a successful *in vivo* study, first of all, it is necessary to develop a tool including a specific antibody, which can discriminate between proHp and mature Hp, and specifically detect only proHp in tissues containing much more mature Hp.

## Supporting information

S1 Fig The effect of Hp2-2 on the gene expression of vimentin and E-cadherin. (TIF)

S1 Raw images Original blot for Figs 1, 3–5. (PDF)
