# Supplementary figures and images for: Prohaptoglobin inhibits the transforming growth factor-β-induced epithelial-to-mesenchymal transition in vitro by increasing Smad1/5 activation and suppressing the Smad2/3 signaling pathway in SK-Hep1 liver cancer cells
Source: PLoS One. 2022 May 17;17(5):e0266409. doi: 10.1371/journal.pone.0266409 (PMC9113573; doi:10.1371/journal.pone.0266409)

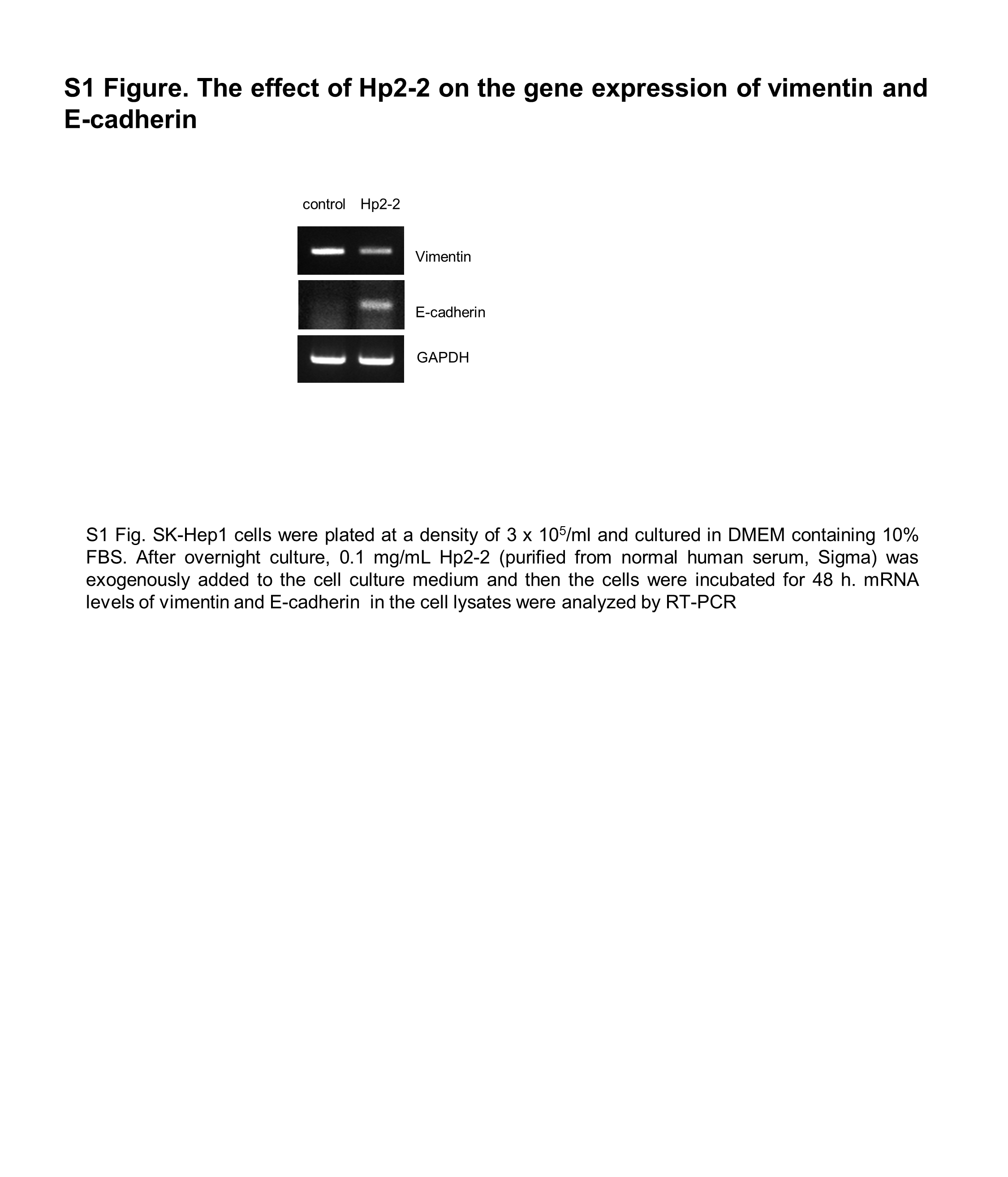

Supplement: S1 Fig — (TIF) [file pone.0266409.s001.TIF]
