# Supplementary material for: Prohaptoglobin inhibits the transforming growth factor-β-induced epithelial-to-mesenchymal transition in vitro by increasing Smad1/5 activation and suppressing the Smad2/3 signaling pathway in SK-Hep1 liver cancer cells
Source: PLoS One. 2022 May 17;17(5):e0266409. doi: 10.1371/journal.pone.0266409 (PMC9113573; doi:10.1371/journal.pone.0266409)

Original image corresponding to Figure 1.

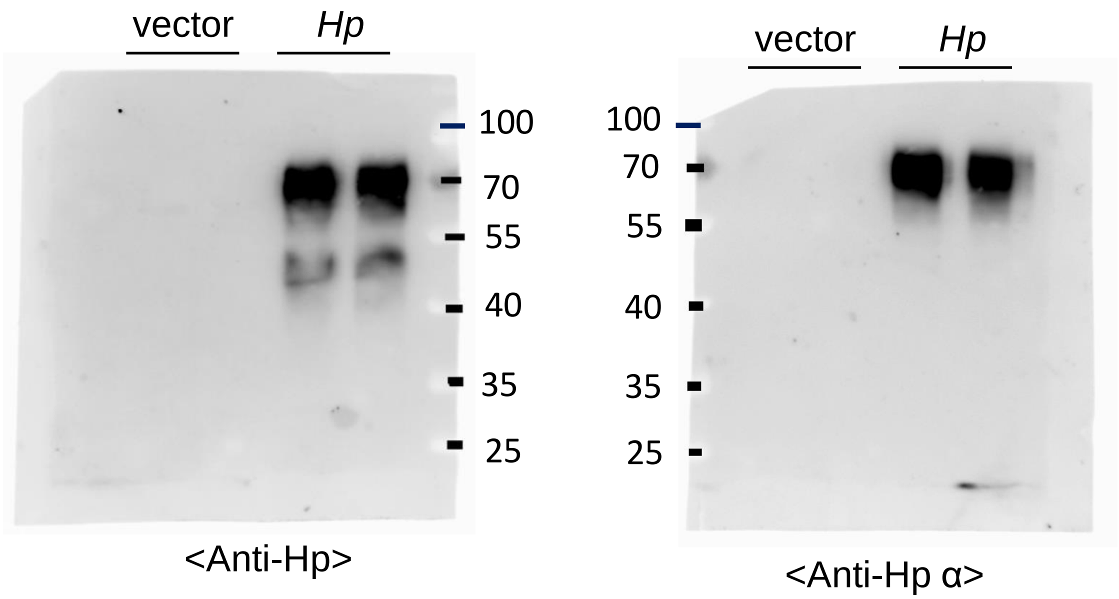

Original image corresponding to Figure 3A.

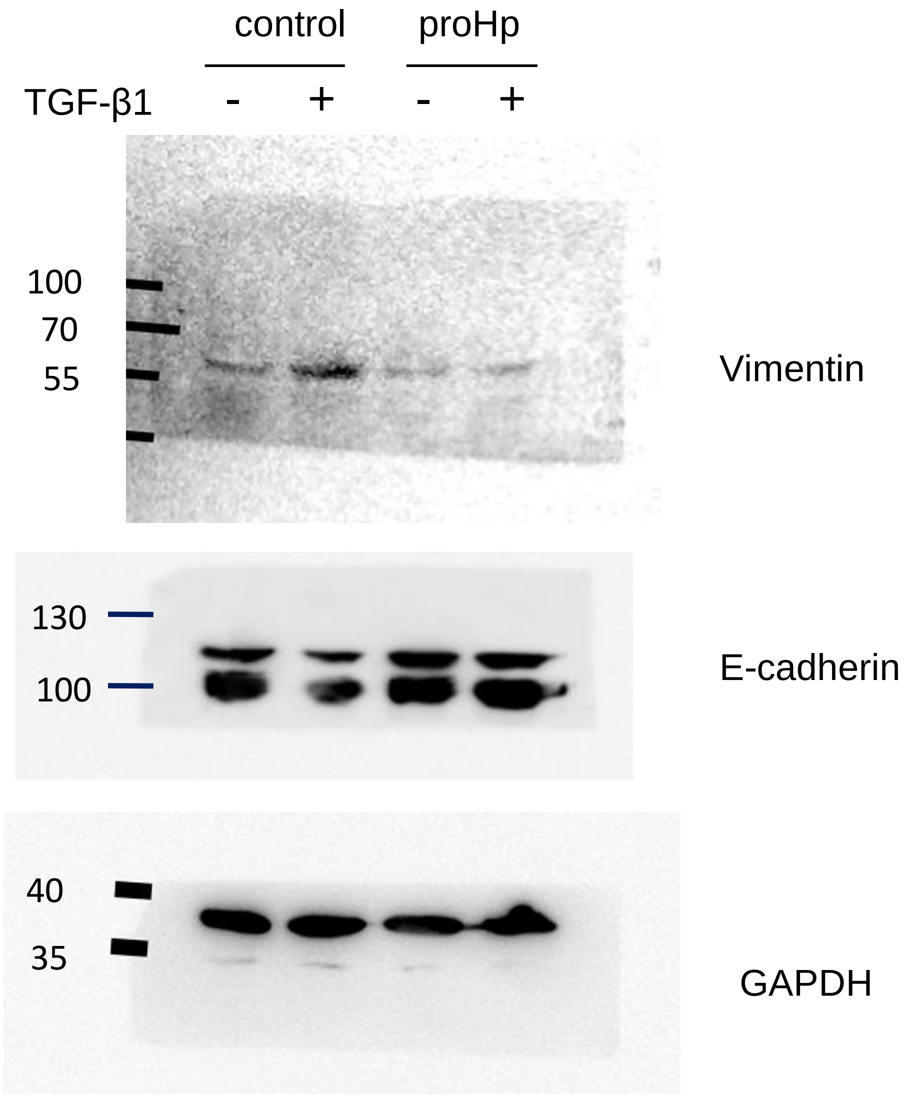

Original image corresponding to Figure 4A.

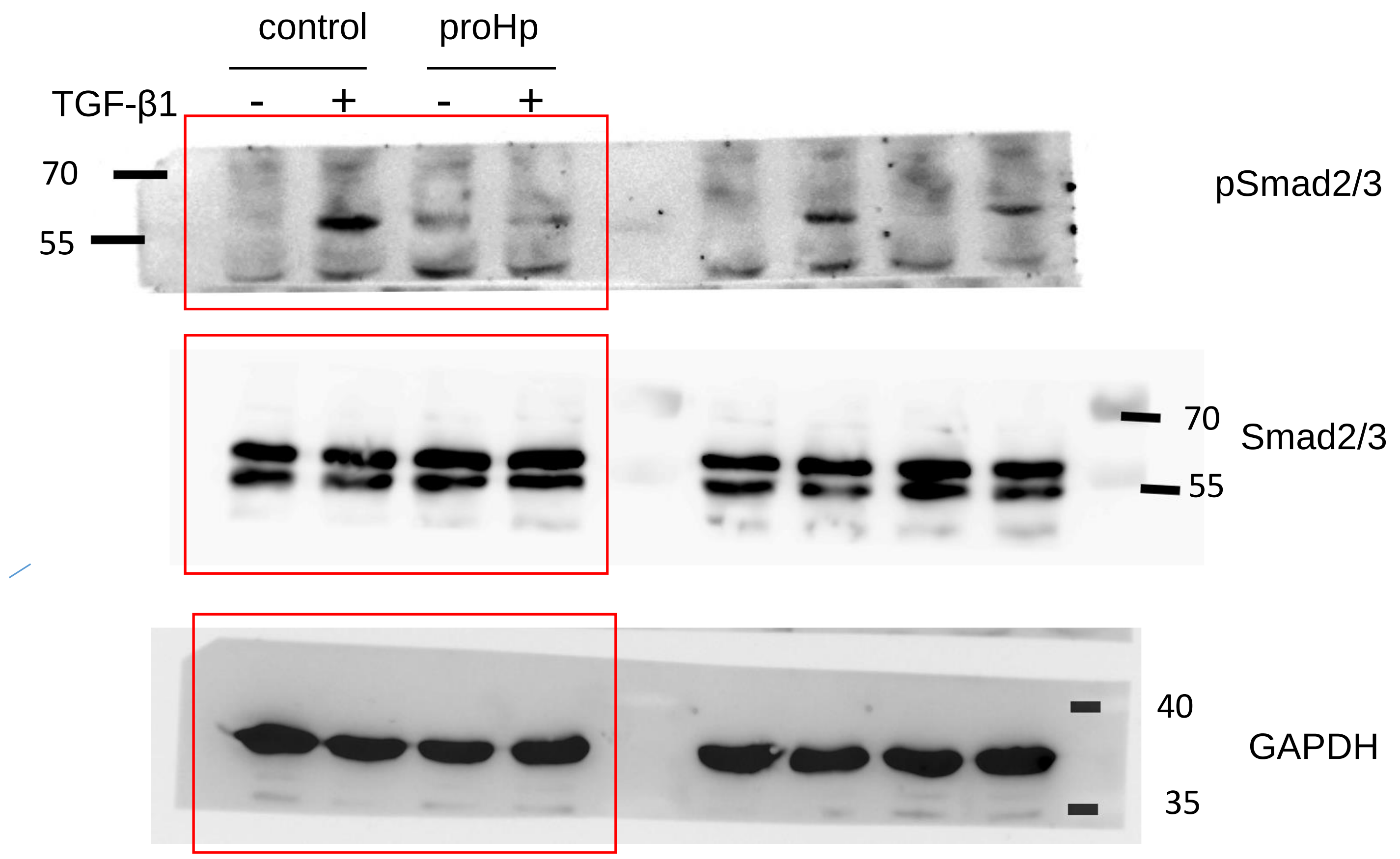

Original image corresponding to Figure 4B.

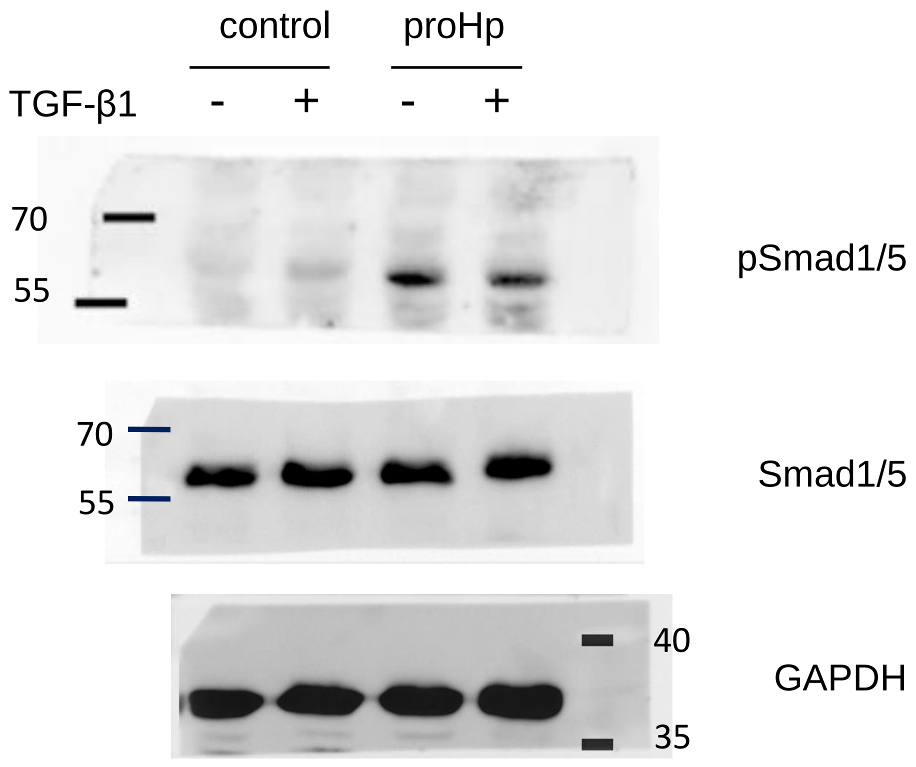

Original image corresponding to Figure 5A.

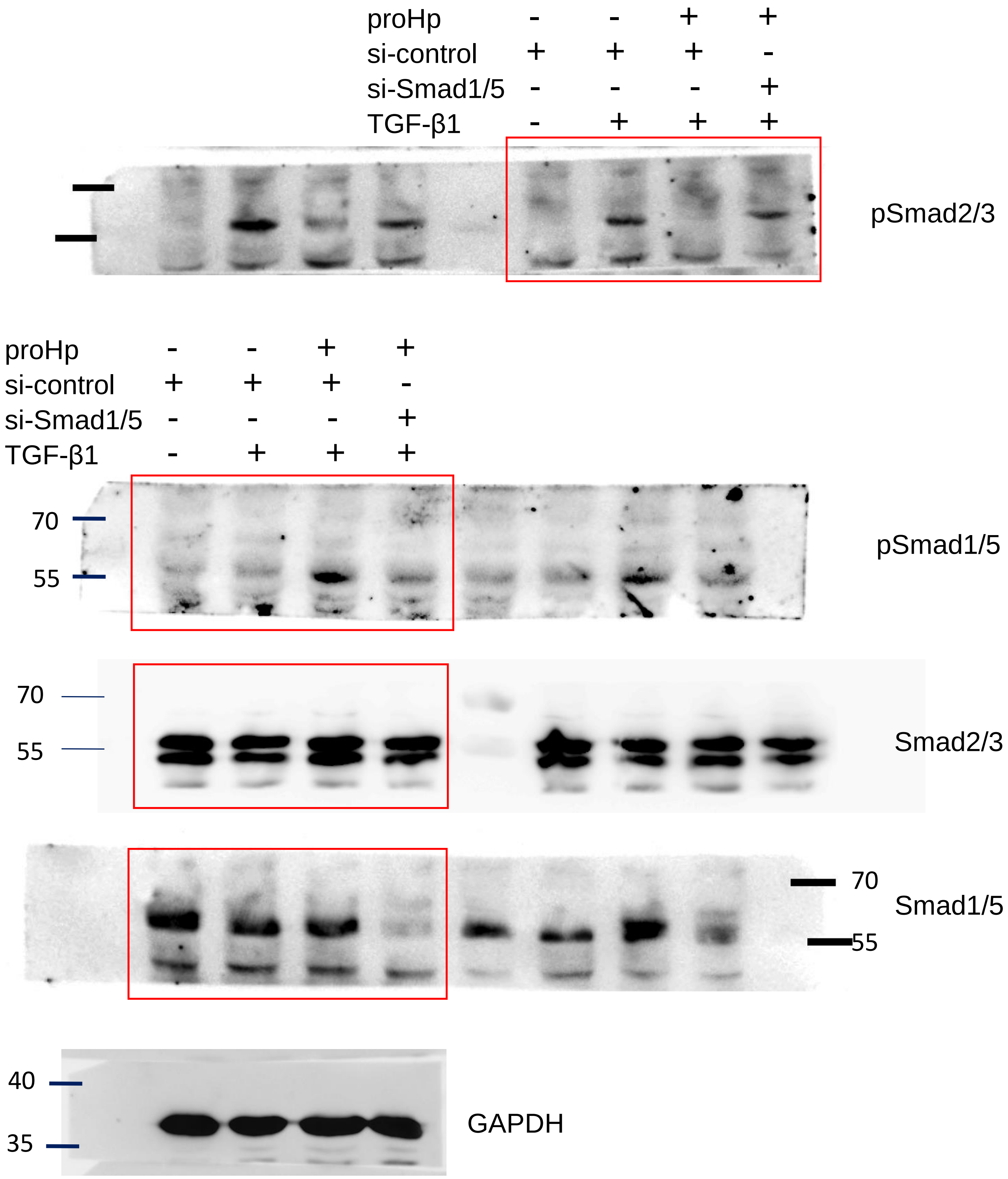

Supplement: S1 Raw images — (PDF) [file pone.0266409.s002.pdf]
